# Development and Validation of an Ultrasound Imaging Algorithm for Structured Reporting in Testicular Pathology

**DOI:** 10.3390/diagnostics15080951

**Published:** 2025-04-09

**Authors:** Roxana Pintican, Alexandru Negrea, Isabell Boll, Bianca Boca, Diana Gherman, Marilena Bora, Sorin Dudea, Anca Ciurea

**Affiliations:** 1 Department of Radiology, “Iuliu Hatieganu” University of Medicine and Pharmacy, 400347 Cluj-Napoca, Romania; isiboll@gmx.net (I.B.); gherman_diana@elearn.umfcluj.ro (D.G.); sdudea1@elearn.umfcluj.ro (S.D.); ancaciurea@elearn.umfcluj.ro (A.C.); 2 Department of Radiology, Prof Dr Ion Chiricuta Oncology Institute, 400015 Cluj-Napoca, Romania; 3 Department of Emergency, County Emergency Hospital, 400347 Cluj-Napoca, Romania; 4 Department of Imaging, “Iuliu Hatieganu” University of Medicine and Pharmacy, 400347 Cluj-Napoca, Romania; petresc.bianca@elearn.umfcluj.ro; 5 Department of Radiology, County Emergency Hospital, 400347 Cluj-Napoca, Romania; marilenageorgiana.bora@gustaveroussy.fr; 6 Department of Radiology, Goustave Roussy Insitute, 94800 Villejuif, France

**Keywords:** testicular, US, diagnostic algorithm, standardized reporting, testicular cancer

## Abstract

**Background/Objectives**: Testicular ultrasound (US) imaging is a critical modality for diagnosing a variety of testicular pathologies, including malignancies. This study aimed to develop and validate a standardized diagnostic algorithm to enhance diagnostic accuracy and consistency in evaluating testicular lesions, particularly for distinguishing between benign and malignant conditions. **Methods**: The algorithm was applied retrospectively to 110 testicular imaging cases, including 90 abnormal and 20 normal cases, analyzed by three radiologists with varying experience levels. Key diagnostic features, including lesion morphology, vascularity, and echotexture, were evaluated to guide the differentiation process. **Results**: demonstrated high diagnostic accuracy, with sensitivity reaching 100% for detecting abnormal cases and specificity ranging between 80% and 95%. In distinguishing benign from malignant lesions, the algorithm achieved an area under the curve (AUC) of up to 0.917, with specificities exceeding 93%. Notably, strong inter-rater agreement was observed, underscoring the algorithm’s reliability across different expertise levels. While the algorithm significantly improved standardization and diagnostic performance, some variability in sensitivity for less experienced evaluators highlights the need for further refinement. **Conclusions**: This study shows that the proposed diagnostic algorithm is an effective tool for testicular US, facilitating accurate and reproducible assessments, which are crucial for early detection and optimal management of testicular pathologies.

## 1. Introduction

Imaging evaluation of the testicles primarily relies on an ultrasound (US), a modality that stands out due to its high diagnostic accuracy, widespread availability, and non-invasive nature [1]. With advancements in technology, the indications for requiring a testicular ultrasound have expanded considerably and now depend on factors such as the patient’s age, clinical symptoms, and medical history. For neonates and infants, a US is most commonly used to confirm the presence of testes in cases of cryptorchidism. In older children, a US is used to evaluate various conditions, including testicular pain, suspected orchitis or epididymitis, testicular or epididymal asymmetry, trauma, torsion, endocrine disorders such as precocious puberty, and abnormal laboratory findings (like elevated alpha-fetoprotein or β-HCG). In adults, a testicular US is essential for evaluating abnormal testicular consistency, suspected tumors, hydrocele, hernias, reproductive failure, and conditions affecting the spermatic cord [1,2,3,4,5].

US imaging, particularly in the context of testicular pathology, not only provides critical diagnostic insights but also plays a significant role in improving long-term outcomes in population-level health screening programs. With advancements in imaging technology, the ability to diagnose subtle pathologies has greatly improved, positioning US imaging as a cornerstone in both acute and preventive care across diverse healthcare systems.

Given the broad spectrum of clinical presentations, it is essential for US image examiners to have a deep understanding of the relevant pathologies and to adhere to standardized imaging protocols and reporting. Such standardization ensures that the results of testicular USs are reproducible and comparable, promoting consistency across different clinical settings and examiners.

However, despite its advantages, testicular US imaging has limitations, particularly its human factor, which can lead to variability in results. The quality of US images can vary significantly based on the examiner’s experience and the equipment used [1,6]. Additionally, in certain cases, such as obesity or scrotal edema, the penetration of sound waves may be hindered, affecting image quality. Thus, achieving consistent and high-quality results requires rigorous standardization and expertise. While the US is the imaging modality of choice for evaluating the scrotum, including the testicles, there is no consensus regarding the technique or diagnostic criteria, making it essential to standardize the examination process and reporting [6,7,8,9].

Testicular cancer, for example, is one of the most prevalent solid tumors among young men, and early detection is crucial for effective treatment. While it typically presents as a solid mass, smaller nodules can be more challenging to diagnose. Nevertheless, prompt identification and treatment of testicular cancer have been shown to result in a 5-year survival rate of 97%, highlighting the importance of early diagnosis for improving patient outcomes [10,11]. Conditions like epididymorchitis, hematomas, and testicular torsion can mimic malignancies, underscoring the importance of accurate diagnosis.

Standardized reporting in US imaging has shown significant benefits in improving clinical outcomes, workflow efficiency, and data consistency. By following structured reporting templates, radiologists can reduce subjectivity and enhance accuracy, leading to more reliable and consistent reports. Structured reporting not only helps in eliminating ambiguities but also streamlines communication between radiologists and referring clinicians, ensuring that reports are clear, comprehensive, and actionable [12,13,14]. The BI-RADS reporting system, widely used in breast imaging, demonstrates how structured reporting enhances diagnostic clarity and fosters consistent communication among healthcare providers [15]. Although structured reporting has been associated with improved diagnostic clarity and reduced ambiguity in imaging interpretation across various modalities, such as breast and prostate imaging, its specific application to testicular US imaging remains underexplored. Studies investigating non-structured reporting in US imaging of the testes typically focus on preliminary feasibility rather than providing substantial evidence of improved clinical outcomes or diagnostic precision [16]. To the best of our knowledge, none of these studies have specifically focused on structured reporting. Furthermore, there is currently no widely adopted consensus guideline identifying the essential elements that should be included in a structured testicular US imaging report, particularly for differentiating benign from malignant lesions, characterizing inflammatory conditions, or evaluating acute scrotal pain. Available guidelines primarily address US imaging equipment requirements, techniques, and common pathology descriptors [1]. This absence of standardized reporting elements results in variability in the quality and completeness of US reports, potentially negatively impacting clinical decisions and patient outcomes.

This highlights a significant gap in research, underscoring the urgent need for studies that develop and validate standardized reporting criteria specifically tailored to testicular US imaging. This study aims to analyze how a standardized US imaging algorithm can assist in providing accurate diagnoses, even for operators with limited experience in testicular US imaging. By implementing such standardization, the diagnostic process can become more efficient and reliable, ultimately improving patient care and outcomes.

## 2. Materials and Methods

### 2.1. Developing the US Imaging Algorithm

The proposed US imaging diagnostic algorithm simplifies the evaluation of testicular pathology into four straightforward steps, employing the mnemonic “4Ss.” The first “S” corresponds to the “site”, determining whether the testis is correctly positioned within the scrotum or located elsewhere, such as in ectopia or cryptorchidism. The second “S” assesses “size”, considering the patient’s age and symmetry in comparison to the contralateral testis. Here, charts showing the size according to the age of the patient are included in the algorithm. The third “S” evaluates the “structure” of the identified abnormality, classifying lesions as cystic, solid, or calcified. For this step, after recognizing and classifying the abnormality, additional characteristics were added to the algorithm, such as cystic, solid, and calcified structures. Cystic lesions included normal variants such as cystic degeneration of the testicular mediastinum, simple cysts, and also complex cysts (like abscesses in tuberculosis or hematomas). The solid tumors were divided between seminomas and non-seminomas, each with US image characteristics and histopathology correlation. Corresponding Doppler and elastography images were presented together with 2D images. Calcifications included cases of vascular calcifications, unifocal, multifocal, and bilateral microlithiasis. The final “S” focuses on “small vessel flow”, using Doppler/Power imaging to assess vascularity and identify conditions like torsion, acute ischemia, or testicular infarction.

The proposed 4S diagnostic framework was meticulously designed following extensive consultations with expert radiologists and iterative refinement based on real-world clinical data. Each step was carefully selected to reflect the most common diagnostic challenges encountered in testicular pathology while ensuring that the approach remains intuitive for general radiologists.

**Figure 1:**
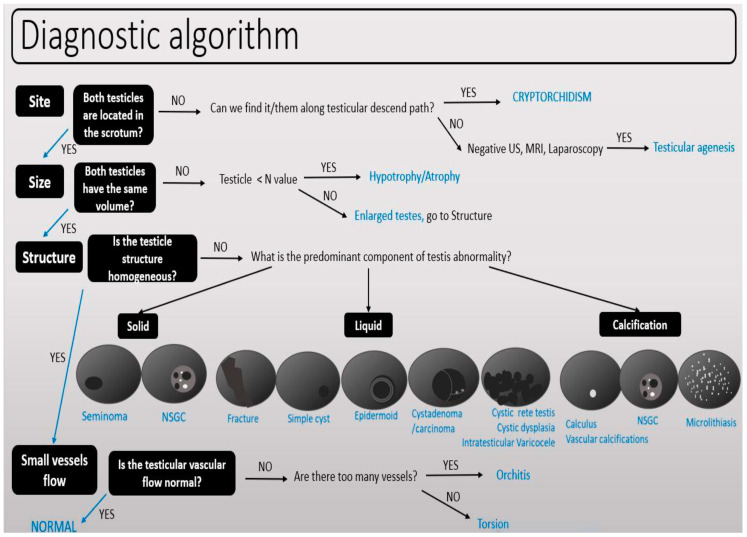
The 4Ss proposed ultrasound imaging algorithm for testicular pathology.

**Figure 2:**
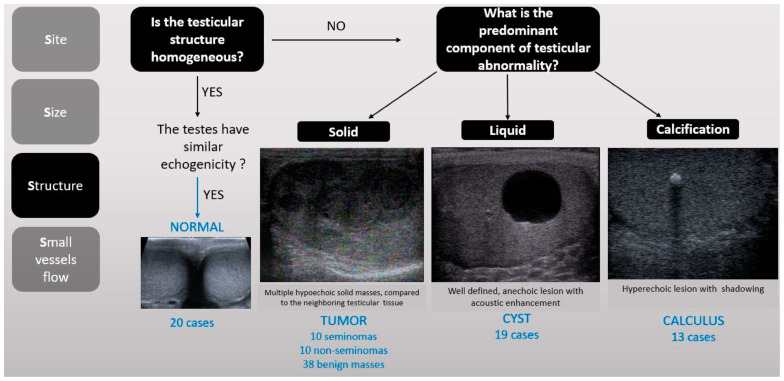
The third “S” stands for structure, referring to the nature of the abnormality. If one testicle exhibits a different echogenicity compared to the contralateral side, a lesion should be suspected. Based on the observed characteristics, lesions may be classified as solid (iso- or hypoechoic), cystic (anechoic), or calcified (hyperechoic).

This comprehensive algorithm was initially presented at the RSNA Annual Meeting in Chicago in 2019, where it earned a Cum Laude award for its innovative approach. The full algorithm, along with detailed guidelines, is accessible through the RSNA EdCentral platform (Appendix A) for further reference and educational purposes [17] (See Figure 1 and Figure 2).

### 2.2. Testing the US Imaging Algorithm

All imaging cases used to evaluate the proposed algorithm were obtained by an experienced radiologist (S.D.), with over 40 years of experience in testicular US imaging and extensive subspecialty training in Europe and the United States. Ultrasound examinations were performed using a Hitachi unit equipped with a high-frequency 7–15 MHz linear transducer. The dataset consisted of 110 cases, including 90 abnormal cases (81.8%, 20 malignant and 70 benign cases) and 20 normal testicular cases (18.2%). The sample size of 110 cases was chosen to balance feasibility with ensuring adequate representation of both common and rare testicular pathologies. Out of 110 cases, 20 were normal, 70 benign (19 cystic, 13 calcified, and 38 solid lesions), and 20 malignant (10 seminomas and 10 non-seminomas cases). There were 11 cases with at least two associated pathologies (e.g., seminoma and microlithiasis). Each standard case contained a minimum of four images, corresponding to the algorithm’s 4 S’s: one for the site (testicular location within the scrotum), a grayscale long/short axis and transverse comparative image of both testicles, and a Doppler Color or Power Doppler image. For pathological cases, additional images were provided, with short video clips included for particularly challenging cases.

Three radiologists, each with different levels of experience, participated in the study: reader 1, a junior resident with one year of general US imaging experience; reader 2, a senior resident with four years of experience in general US imaging; and reader 3, a board-certified radiologist with six years of general US imaging practice. None of the readers had prior experience with testicular or scrotal US imaging.

**Figure 3:**
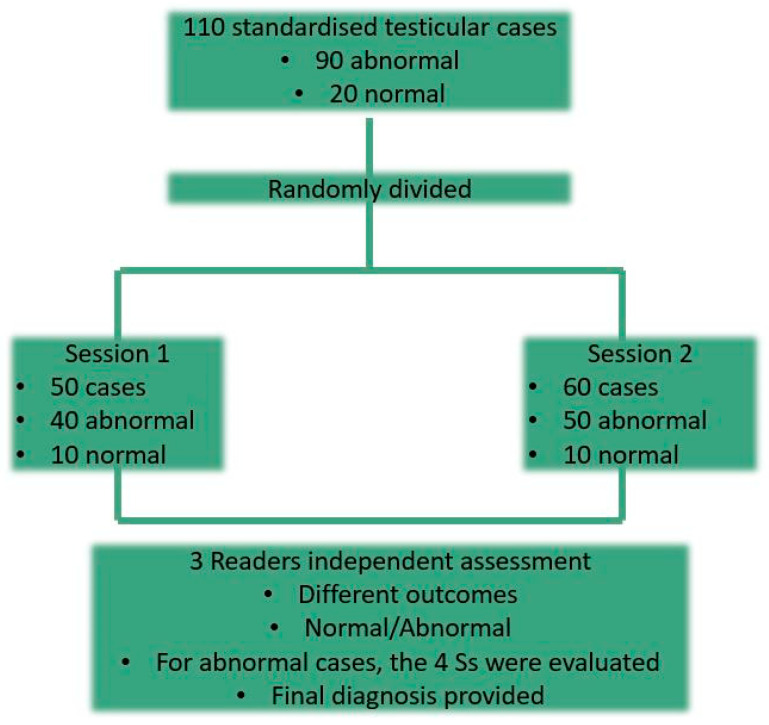
The flow chart showing how the algorithm was tested by the readers.

The algorithm was assessed through a retrospective multi-reader study conducted between December 2022 and February 2023, involving two randomized testing sessions to minimize bias. The first session included 50 cases, and the second 60, each lasting approximately 90 min. Readers were introduced to the algorithm through a PowerPoint presentation that was available one month before the testing sessions, which aimed to familiarize them with testicular US imaging terminology and interpretation techniques. Readers were blinded to the total number of images per case and the final pathology to ensure unbiased evaluation (See Figure 3).

Using the proposed algorithm, the three readers were tasked with answering six key diagnostic questions for each case: (1) Is the testicle’s location normal? (2) Is the testicle itself normal or abnormal? (3) If abnormal, is the lesion benign or malignant? (4) Does the lesion appear solid, cystic, or calcified? (5) Are there any vascularity changes observed using Doppler US? (6) What is the final diagnosis? These questions ensured a systematic and thorough evaluation of each testicular US imaging case, guided by the algorithm’s structured framework. We divided the primary endpoint between accuracy in identifying malignant cases (question number 3) and secondary endpoints (for all the remaining questions).

Additionally, each case was rated for difficulty on a scale of 1 to 3, with 1 representing straightforward, easy cases, 2 moderately challenging cases, and 3 the most complex cases. This classification provided insight into the algorithm’s performance across varying levels of diagnostic complexity, reflecting its adaptability and reliability in both simple and challenging clinical scenarios.

To ensure rigorous evaluation, the algorithm was tested using a randomized multi-reader study design, which accounted for both clinical complexity and operator experience. This robust methodology provided a comprehensive assessment of its diagnostic accuracy across diverse scenarios.

### 2.3. Statistical Analysis

To assess the diagnostic accuracy of the proposed US imaging algorithm, sensitivity, specificity, positive predictive value (PPV), negative predictive value (NPV), and the area under the receiver operating characteristic curve (AUC-ROC) were analyzed for each reader, and compared to the gold standard (pathology results or long-term follow up of >5 years).

Inter-rater reliability was assessed using Cohen’s kappa coefficient to evaluate the consistency of interpretations among three readers with varying levels of expertise. Kappa values were interpreted as follows: <0.2 poor, 0.21–0.4 fair, 0.41–0.6 moderate, 0.61–0.8 substantial, >0.8 near-perfect agreement. This helped us determine the reproducibility of the algorithm across different operators. Several subgroup analyses were performed to investigate the algorithm’s performance across patient groups categorized by pathology type (e.g., tumors, microlithiasis) and clinical variables. Statistical significance was determined using appropriate tests (e.g., chi-square tests for categorical data and *t*-tests for continuous variables), with 95% confidence intervals (CIs) alongside the *p*-values to provide an understanding of the precision and reliability of these estimates. A threshold of *p* < 0.05 was used to indicate statistical significance. All the statistical analyses were performed using MedCalc, available online at www.medcalc.org (accessed on 17 December 2024).

## 3. Results

### 3.1. Primary Endpoint Assessment by the Three Readers

**Figure 4:**
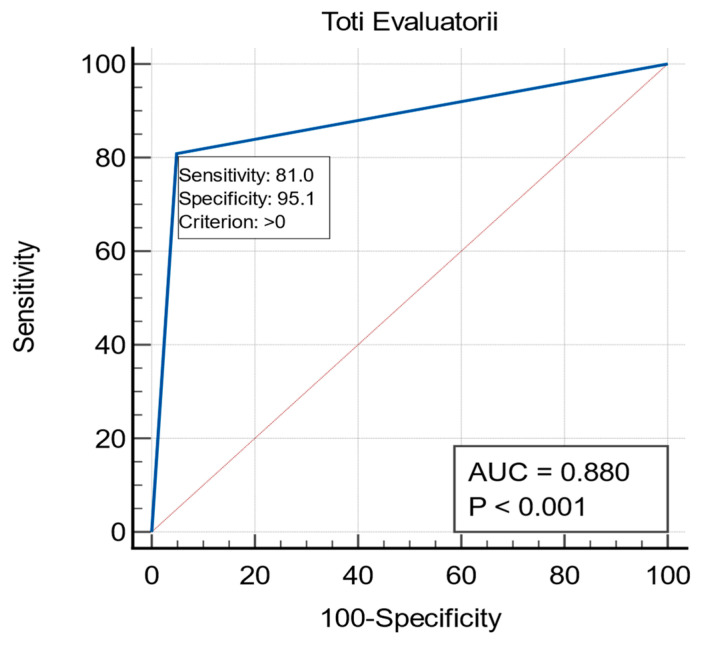
AUC, Sensitivity, and Specificity for differentiating benign versus malignant testicular cases.

First, we assessed the algorithm’s ability to differentiate between benign and malignant pathologies, and observed AUC values that ranged from 0.823 to 0.917. Sensitivity varied between 71.43% and 85.71%, while specificity was higher, ranging from 93.26% to 97.75%. These findings illustrate that while the algorithm performed well in distinguishing malignant from benign conditions, some variability was observed in sensitivity, particularly for reader 2 (See Figure 4).

When assessing seminoma cases compared to other pathological findings, the AUC values slightly increased, ranging from 0.841 to 0.923. Specificity in this category was exceptionally high, with Reader 3 achieving 100%. Sensitivity ranged from 69.23% to 84.62%, highlighting the algorithm’s capability to identify malignancy (See Table 1).

Inter-rater agreement, as measured by Cohen’s kappa coefficient, demonstrated strong consistency among the three readers. For distinguishing normal from pathological cases, kappa values exceeded 0.8 across all comparisons, indicating a high degree of reliability. Agreement was similarly strong in identifying benign versus malignant pathologies, with kappa coefficients ranging from 0.772 to 0.856. These findings confirm that the algorithm promotes a standardized approach, reducing variability in diagnostic outcomes regardless of the evaluator’s experience (See Table 2).

### 3.2. Secondary Endpoints Assessment by the Three Readers

**Figure 5:**
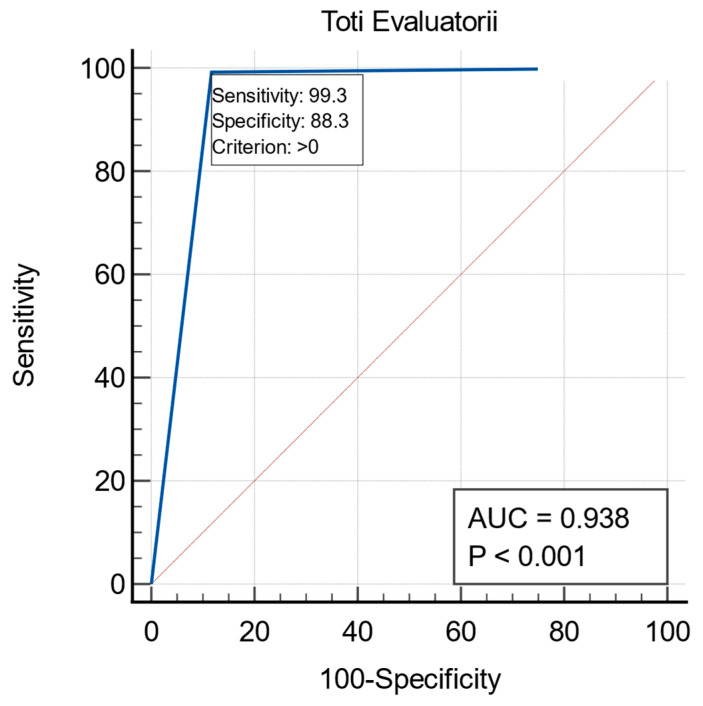
AUC, Sensitivity, and Specificity for differentiating normal versus abnormal testicular cases.

The proposed diagnostic algorithm demonstrated robust performance across several testicular pathologies, as evaluated by three radiologists with varying levels of expertise. When distinguishing normal from abnormal pathological cases, the algorithm exhibited high diagnostic accuracy. The AUC values, which quantify the overall diagnostic ability, ranged from 0.900 to 0.975 among the three evaluators. Sensitivity, a measure of the ability to correctly identify pathological cases, was 100% for two of the evaluators, while the third achieved 97.78%. Specificity, which reflects the ability to correctly identify normal cases, ranged between 80% and 95%, indicating strong reliability in excluding false positives (See Table 1 and Figure 5).

For the cystic, solid, and mixed lesion classification, interobserver agreement was moderate, with kappa values ranging from 0.432 to 0.550 and an average of 0.478. When compared to the gold standard, agreement was lower, with kappa values of 0.300, 0.267, and 0.184, leading to an average kappa of 0.250, which reflects fair agreement at best. This disparity suggests notable variability among evaluators and between evaluators and the gold standard, highlighting the potential subjectivity in assessing these lesion types.

In the cystic versus non-cystic classification, performance metrics varied among evaluators. Evaluator 1 demonstrated high sensitivity, specificity, and AUC (0.80, 0.89, and 0.854, respectively), indicating robust performance in identifying cystic lesions. Evaluator 2, however, exhibited low sensitivity at 0.20 but maintained high specificity at 0.91 and a moderate AUC of 0.716. Evaluator 3 achieved more balanced metrics, with a sensitivity of 0.71, specificity of 0.92, and AUC of 0.817, reflecting strong overall diagnostic ability. These discrepancies point to differences in evaluators’ ability to consistently classify cystic lesions, underscoring the need for standardization.

In the mixed solid-cystic lesions versus non-mixed lesions (either cystic or solid) classification, specificity was relatively high for all evaluators, ranging from 0.86 to 0.89, but the AUC values, which ranged from 0.468 to 0.597, indicate limited overall diagnostic effectiveness.

The evaluation of microlithiasis yielded similarly favorable outcomes. AUC values for differentiating microlithiasis from other pathologies ranged from 0.901 to 0.941, with sensitivity and specificity consistently exceeding 88% and 95%, respectively. In one notable case of microlithiasis with an unusual vascular pattern, all three readers demonstrated consistent diagnostic accuracy using the 4S algorithm, underscoring its robustness even in atypical presentations. However, the 95% specificity suggests that the algorithm is highly reliable for identifying microlithiasis (See Table 1).

The assessment of small vessel flow showed a substantial agreement between readers, with kappa values ranging from 0.632 to 0.780 and an average of 0.718. Agreement with the gold standard was also strong, with kappa values ranging from 0.536 to 0.660 and an average of 0.617. Performance metrics reflected robust diagnostic accuracy, with Evaluator 1 achieving a sensitivity of 0.74, specificity of 0.97, and AUC of 0.860. Evaluator 2 recorded a sensitivity of 0.61, perfect specificity at 1.00, and an AUC of 0.809. Evaluator 3 exhibited a sensitivity of 0.73, specificity of 1.00, and an AUC of 0.866. These results demonstrate that small vessel flow assessment was the most reliable and diagnostically effective category.

In terms of overall diagnostic accuracy, interobserver agreement was fair, with kappa values ranging from 0.342 to 0.404 and an average of 0.370. Evaluators demonstrated high accuracy, with overall rates of 84.5% for Evaluator 1, 86.4% for Evaluator 2, and 87.3% for Evaluator 3. Although the diagnostic accuracy was high, the fair level of agreement suggests variability in interpretation among evaluators.

**Figure 6:**
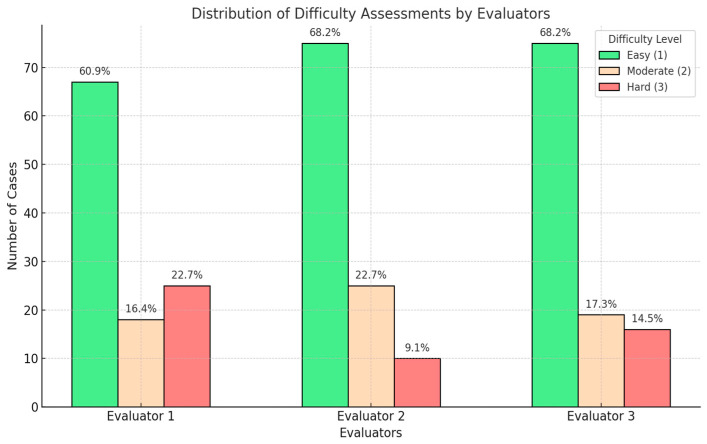
The distribution of the cases, assessed as easy, moderate, or difficult from the difficulty point of view, of the three readers.

The analysis of difficulty levels revealed low interobserver agreement, with an average kappa of 0.166 between evaluators and 0.259 with the gold standard. Accuracy was highest for easy cases, with scores of 96.6% for Evaluator 1, 94.9% for Evaluator 2, and 93.2% for Evaluator 3. For moderate cases, accuracy declined to 65% for Evaluator 1 and Evaluator 3, and 75% for Evaluator 2. In difficult cases, Evaluator 3 outperformed the others with an accuracy of 90.3%, compared to 74.2% for Evaluator 1 and 77.4% for Evaluator 2. (See Figure 6 and Table 3).

## 4. Discussion

The findings underscore the effectiveness of the proposed diagnostic algorithm in standardizing the evaluation of testicular USs across readers with varying levels of experience. Its performance in differentiating benign from malignant cases was particularly notable, with high sensitivity and specificity values demonstrating its reliability in accurately identifying benign from malignant pathology. This suggests that the algorithm provides a robust framework for clinicians, including those with limited experience in testicular USs, to detect significant pathologies.

While several studies emphasize the value of standardized reporting in US imaging [18,19,20], few have specifically evaluated its role in testicular imaging, and most focus on only one pathology [21,22]. No comprehensive studies have assessed standardized reporting across both benign and malignant testicular diseases, nor have definitive protocols been proposed. The currently suggested 4Ss algorithm is both easy to recall and highly effective, even when applied by readers with diverse levels of expertise. However, its ability to distinguish between benign and malignant conditions, while robust, revealed some limitations in sensitivity. Variability in performance, particularly noted in Reader 2, may reflect the small sample size of raters and highlights the need for further studies with larger cohorts.

Small vessel flow assessment stood out as the most reliable and diagnostically effective area, with high interobserver agreement and strong performance metrics. In contrast, the assessment of cystic, solid, or mixed solid lesions showed significant challenges, with low sensitivity and poor agreement, even if the overall diagnostic accuracy was high. One explanation could be in the terminology, for example, a dermoid cyst was classified as a cystic lesion by one reader, while the other categorized the lesion as solid. This indicates that our algorithm is better suited for distinguishing between benign and malignant pathology rather than providing a comprehensive and detailed characterization of individual lesions.

The algorithm also proved effective in identifying microlithiasis, a condition associated with an increased risk of testicular malignancy. Its high sensitivity and specificity suggest the tool reliably identifies patients requiring closer surveillance or cancer screening [23,24]. Additionally, its high specificity in identifying seminomas supports its utility in distinguishing certain malignancies, though moderate sensitivity in some cases indicates the need for supplementary imaging techniques, such as multiparametric US, in ambiguous scenarios.

The decline in diagnostic accuracy with increasing case complexity underscores the necessity for advanced diagnostic aids to assist evaluators in intricate scenarios. While certain aspects of testicular US imaging evaluation were robust, others require targeted improvements to ensure consistency and reliability. For instance, cases classified as difficult often involve multiple associated pathologies, including exceptionally rare synchronous cancers of different histology. Cicero et al. [25] highlighted that multiple focal lesions identified at imaging within the testis are not always of the same histology, emphasizing the need for careful evaluation in such complex cases.

Recent multicenter studies have emphasized the role of US imaging in diagnosing these complex cases and the necessity for additional training. Santos et al. [26] conducted a multicenter retrospective study on benign testicular tumors in children, aiming to describe the incidence, histology, and surgical techniques, with a special emphasis on approaches that could present better outcomes. These findings suggest that while a US is a valuable tool in the evaluation of testicular lesions, there is a pressing need for enhanced training and diagnostic aids to improve accuracy in complex scenarios [27].

Emerging technologies, including contrast-enhanced ultrasound (CEUS) and elastography, could complement the algorithm by enhancing vascularity assessments, especially in diagnostically challenging cases. Studies of these techniques support their integration into a comprehensive evaluation framework [26,28,29,30,31].

Comparison with existing protocols, such as the FAST protocol in emergency settings, highlights the algorithm’s broader clinical potential [32]. While FAST addresses acute diagnostic needs, the 4Ss algorithm provides a focused approach to testicular pathologies, emphasizing simplicity, accuracy, and early detection. It may also prove valuable in testicular emergencies.

The algorithm demonstrated excellent inter-rater agreement, with kappa coefficients exceeding 0.8 across readers, underscoring its potential to reduce variability in diagnostic outcomes. Consistency across evaluators, regardless of experience, is critical in clinical practice, where accurate US imaging assessments significantly impact patient outcomes.

Despite its strengths, the study has notable limitations. Its retrospective design precludes consideration of real-time patient factors, which can affect image quality and diagnostic accuracy. The relatively small sample size also limited the ability to perform more detailed subgroup analyses, such as for ischemia. Incorporating an abdominal ultrasound in future studies would enhance diagnostic completeness, particularly in cases where scrotal symptoms originate from abdominal pathology. Prospective studies may assess the algorithm in real-time practice, on a larger cohort of patients, towards a more robust validation of the algorithm. Emerging trends in imaging modalities, such as contrast-enhanced US and elastography, promise to address some of these limitations. These technologies enable enhanced visualization of vascularity and tissue elasticity, providing additional layers of diagnostic information for complex cases.

As future directions, structured reporting systems, such as this algorithm, also support data mining and applications of artificial intelligence (AI). By enabling efficient data aggregation and analysis, such tools can enhance research, inform population health initiatives, and improve patient care through clearer, standardized clinical reporting. Furthermore, the integration of AI into the 4Ss algorithm may represent a promising avenue for future research. Automated pattern recognition and decision support systems could enhance the reproducibility and accessibility of this diagnostic tool, particularly in settings where experienced radiologists are unavailable.

## 5. Conclusions

The 4Ss diagnostic algorithm demonstrated substantial benefits in standardizing testicular ultrasound (US) imaging assessments, leading to improved diagnostic accuracy, sensitivity, and specificity. The results indicated that even less experienced readers can reliably identify testicular malignancies using this structured approach. Implementing the 4Ss algorithm has the potential to enhance early detection and treatment outcomes, particularly in healthcare settings with limited expertise in testicular US.

## Figures and Tables

**Table 1:** The AUC, sensitivity, and specificity of the proposed US imaging algorithm for different scenarios.

Scenario	AUC	Sensitivity %	Specificity %
Benign vs. Malignant			
R1	0.9	85.71	94.38
R2	0.82	71.43	93.26
R3	0.91	85.71	97.75
R1 + R2 + R3	0.88	80.95	95.13
Seminoma vs. All pathology			
R1	0.87	76.92	97.94
R2	0.84	69.23	98.97
R3	0.92	84.62	100
Normal vs. Abnormal			
R1 + R2 + R3	0.93	99.26	88.33
Microlithiasis			
R1 + R2 + R3	0.94	88	95

R1 = reader one, junior resident; R2 = reader two, senior resident; R3 = board-certified radiologist. AUC = area under the curve.

**Table 2:** Inter-reader agreement coefficients regarding the differentiation between benign and malignant cases.

Scenario	Inter-Reader Agreement (Kappa Coefficients)
Reader 1 vs. Gold Standard	0.77284
Reader 1 vs. Gold Standard	0.64687
Reader 1 vs. Gold Standard	0.85014
Reader 1 vs. Reader 2	0.84419
Reader 1 vs. Reader 3	0.83035
Reader 2 vs. Reader 3	0.80120

**Table 3:** Overall diagnostic accuracy achieved by the readers.

Reader	AUC for Easy Cases	AUC for Moderate Cases	AUC for Difficult Cases
Reader 1	96.6	65	74.2
Reader 2	94.9	75	77.4
Reader 3	93.2	65	90.3

## Data Availability

The original contributions presented in this study are included in the article. Further inquiries can be directed to the corresponding author.

## Appendix A

The full algorithm was presented at the RSNA 2020, and it is available online through the RSNA EdCentral platform.
